# Green synthesis of nanoparticles with extracellular and intracellular extracts of basidiomycetes

**DOI:** 10.7717/peerj.5237

**Published:** 2018-07-20

**Authors:** Elena Vetchinkina, Ekaterina Loshchinina, Maria Kupryashina, Andrey Burov, Timofey Pylaev, Valentina Nikitina

**Affiliations:** Institute of Biochemistry and Physiology of Plants and Microorganisms, Russian Academy of Sciences, Saratov, Russian Federation

**Keywords:** Green synthesis, Nanoparticles, Xylotrophic basidiomycetes, Extra- and intracellular extracts, Morphogenetic stages, Phenol oxidase

## Abstract

Au, Ag, Se, and Si nanoparticles were synthesized from aqueous solutions of HAuCl_4_, AgNO_3_, Na_2_SeO_3_, and Na_2_SiO_3_ with extra- and intracellular extracts from the xylotrophic basidiomycetes *Pleurotus ostreatus*, *Lentinus edodes*, *Ganoderma lucidum*, and *Grifola frondosa*. The shape, size, and aggregation properties of the nanoparticles depended both on the fungal species and on the extract type. The bioreduction of the metal-containing compounds and the formation rate of Au and Ag nanoparticles depended directly on the phenol oxidase activity of the fungal extracts used. The biofabrication of Se and Si nanoparticles did not depend on phenol oxidase activity. When we used mycelial extracts from different fungal morphological structures, we succeeded in obtaining nanoparticles of differing shapes and sizes. The cytotoxicity of the noble metal nanoparticles, which are widely used in biomedicine, was evaluated on the HeLa and Vero cell lines. The cytotoxicity of the Au nanoparticles was negligible in a broad concentration range (1–100 µg/mL), whereas the Ag nanoparticles were nontoxic only when used between 1 and 10 µg/mL.

## Introduction

Nanoparticles have unique catalytic, electronic, magnetic, chemical, photoelectrochemical, and optical properties and are important in technology and medicine. Au nanoparticles are highly stable, low reactogenic, and biocompatible. They generally lack specific toxicity, come in a variety of shapes, have unique optical and electronic properties, and can be used in optics, electronics, catalysis, and biomedicine (diagnostics, therapy of cancer and other diseases, and drug and gene delivery) (Daniel & Astruc, 2004; Chauhan et al., 2011;  Austin et al., 2014; Shah, Badwaik & Dakshinamurthy, 2014; Sherwani et al., 2015; Versiani et al., 2016). Ag nanoparticles, owing to their unique physical, chemical, and biological properties, are effective in chemical catalysis, optoelectronics, biomedicine, and other fields (Prabhu & Poulose, 2012;  Austin et al., 2014; Alarcon, Griffth & Udekwu, 2015; Wei et al., 2015). Ag nanoparticles have antiviral, antifungal, antibacterial, antitumor, anti-inflammatory, and antioxidant properties, which open extensive possibilities for the use of nano-Ag in biomedicine to treat infections and cancers, as well as to prepare medical devices, advanced-therapy medicinal products, and cosmetics (Prabhu & Poulose, 2012; Alarcon, Griffth & Udekwu, 2015; Wei et al., 2015). Mesoporous and nonporous SiO_2_ nanoparticles can be used in such areas of nanobiomedicine as bioimaging and diagnosis, photodynamic therapy, gene and drug delivery, disease targeting, detection of single molecules, and separation and purification of cells and biomolecules (Wang, Zhao & Tan, 2008; Wu & Yamauchi, 2012; Tang & Cheng, 2013). Se nanoparticles are used in the production of rectifiers, solar cells, photocopiers, and semiconductors (Husen & Siddiqi, 2014). Nanoparticles of elementary Se are more biocompatible and less toxic than selenite and selenate. Their biological activity includes antimicrobial, antioxidant, and anticancer properties, which opens broad possibilities for their use in biology and medicine, including the use as nutritional supplements (Wadhwani et al., 2016; Skalickova et al., 2017).

The physicochemical properties, biological activity, and degree of toxicity of nanoparticles depend on their size and shape ( Peng et al., 2007; Kelly et al., 2003; Gatoo et al., 2014; Kulkarni & Muddapur, 2014; Ivask et al., 2014; Shang, Nienhaus & Nienhaus, 2014), and this dependence calls for novel procedures to prepare nanoparticles with required properties and characteristics. Recent years have seen increased use of the simple and eco-friendly synthesis of nanoparticles with living cultures of bacteria, fungi, plants, and algae, as well as with their biomass, extracts, and metabolites (Narayanan & Sakthivel, 2011; Kharissova et al., 2013; Malik et al., 2014; Mishra et al., 2014; Mittal, Chisti & Banerjee, 2013; Ahmed et al., 2016a; Ahmed et al., 2016b; Annu et al., 2018).

Most well-known species of edible mushrooms belong to the group *Basidiomycota* (Cunha Zied & Pardo-Giménez, 2017). In addition, all cultivated species of economic importance are basidiomycetes, including such genera as *Pleurotus*, *Lentinus*, *Grifola*, and *Ganoderma*. Fungi of other groups, such as *Ascomycota* and *Zygomycota*, can synthesize nanoparticles, too (Das et al., 2012; Castro, Cottet & Castillo, 2014; Al Juraifani & Ghazwani, 2015; Baharvandi, Soleimani & Zamani, 2015; Sathishkumar et al., 2015; Sarkar & Acharya, 2017). Many of those fungi, however, are human, animal, or plant pathogens and can induce human allergies. Therefore, they are unfavorable objects to use in biotechnological processes, as compared with edible cultivated basidiomycetes. On the other hand, edible and medicinal cultivated basidiomycetes are promising for biosynthesis of nanoparticles. They are grown in pure culture, are not toxic or pathogenic, and produce a broad range of active protein molecules. In addition, they have potent enzyme systems, which allow them to convert various chemical compounds to less toxic forms, produce copious biomass, and accumulate nanoparticles in their mycelia and culture media (Hulkoti & Taranath, 2014; Dhillon et al., 2012;  Kitching, Ramani & Marsili, 2015; Vetchinkina et al., 2017).

Nanoparticle synthesis with basidiomycetes is still understudied, as compared to synthesis with lower fungi and bacteria, and has begun to be reported only recently. Most studies have used fungal cultures in vivo; however, such cultures contain a variety of unidentified enzymes and other substances, and the results are often irreproducible. Furthermore, no comparisons have been made of the abilities of fungi or their metabolites to effectively make homogeneous, stable nanoparticles of required chemical composition, shape, and size.

Here we examined how culture liquid filtrates and intracellular extracts from the basidiomycetes *P. ostreatus*, *L. edodes*, *G. lucidum*, and *G. frondosa* can be used to synthesize Au, Ag, Se, and Si nanoparticles in vitro. Nanoparticle synthesis with extracts from different stages of fungal development was also studied.

## Materials and Methods

### Fungi and culture conditions

*Pleurotus ostreatus* (Fr.) Kumm. HK-35 (oyster mushroom), *Lentinus edodes* (Berk.) Sing F-249 (shiitake), *Grifola frondosa* (Fr.) S.F. Gray 0917 (maitake), and *Ganoderma lucidum* (Curtis: Fr.) P. Karst 1315 (reishi) were obtained from the Basidiomycetes Culture Collection of Komarov Botanical Institute Russian Academy of Sciences, and from the Collection of Higher Basidial Fungi, Department of Mycology and Algology, Lomonosov Moscow State University. The fungi were maintained on beer-wort agar plates at 4 °C in the higher fungi collection of the Laboratory of Microbiology, Institute of Biochemistry and Physiology of Plants and Microorganisms, Russian Academy of Sciences.

The fungi were grown submerged in a synthetic medium of the following composition (g/L): d-glucose, 1; l-asparagine, 0.1; KH_2_PO_4_, 2; K_2_HPO_4_, 3; MgSO_4_ × 7H_2_O, 2.5; FeSO_4_ × 7H_2_O, 0.03 (PH 5.8). Growth was at 26 °C for 21 days in 100-mL flasks containing 50 mL of the medium. Morphogenetic structures were obtained by growing the fungi on a wood substrate in the laboratory under conditions closest to natural (Vetchinkina & Nikitina, 2007). The structures used were nonpigmented mycelium (NM), brown mycelial mat (BMM), primordia (PR), and fruiting bodies (FB).

### Conditions for preparation of extracts

The culture liquid was separated from the mycelium by centrifugation and was filtered. For intracellular extracts, mycelia or individual morphological structures were separated from the culture medium, rinsed in distilled water, and mechanically ground at 18 °C in a porcelain mortar with quartz sand to destroy the cell envelope. Next, they were extracted with 20 mM Na/K-phosphate buffer (pH 6.0), centrifuged at 12,000 g for 20 min, and filtered. The supernatant liquids were dialyzed against water and were used in the experiments.

For bioreduction studies, the filtrates of the culture liquids and the aqueous intracellular extracts were incubated in the dark at room temperature with aqueous solutions of silver nitrate (AgNO_3_, ≥99.0%), sodium selenite (Na_2_SeO_3_, ≥98.0%), sodium silicate (Na_2_SiO_3_, ≥98.0%) at 0.5 mM, and chloroauric acid (HAuCl_4_, ASC reagent) at 50 µM final concentrations, respectively. All chemicals were from Sigma-Aldrich, St. Louis, MO, USA. Previously, we selected optimal concentrations of these compounds, at which intensive formation of stable nanoparticle colloids took place (Vetchinkina et al., 2017). Solutions of the compounds were added to each sample under sterile conditions. The incubation time needed for nanoparticles formation varied from several minutes to several hours, depending on the used compound and extract.

### Phenol oxidase activity measurements

Enzyme activities were measured with a Specord M40 spectrophotometer (Carl Zeiss, Jena, Germany) in 1-cm path-length quartz cuvettes at 18 °C. Laccase activity was measured at 436 nm by the oxidation rate for 2,2′-azino-bis-(3-ethylbenzothiazoline-6-sulfonic acid) diammonium salt (ABTS; Sigma-Aldrich, USA) (Slomczynski, Nakas & Tanenbaum, 1995). Tyrosinase activity was measured at 475 nm by the oxidation rate for 3-(3,4-dihydroxyphenyl)-l-alanine (l-DOPA; Serva, Germany) (Pomerantz & Murthy, 1974). Mn-peroxidase activity was measured at 468 nm by the oxidation rate for 2,6-dimethoxyphenol (DMOP; Sigma) (Paszczynski, Crawford & Huynh, 1988). Protein was estimated by the Bradford method (Bradford, 1976).

### X-ray fluorescence and X-ray diffraction analysis

The biologically formed nanoparticles were filtered through a Millipore membrane filter (pore size, 0.45 µm), resuspended in minimal distilled water, and air dried at 20 °C. The contents of Au, Ag, Se, and Si were analyzed with an ED 2000 energy-dispersive spectrometer (Oxford Instruments, Abingdon, UK) by using the basic parameters method included in the instrument’s software. The oxidation states of the elements were examined by X-ray diffraction analysis of nanoparticle suspensions. The analysis was conducted with a DRON-3.0 diffractometer (CuKα irradiation; wavelength, 1.54173 Å), by using the JCPDS powder diffraction database (USA 1987).

### Transmission electron microscopy (TEM) and selected-area electron diffraction (SAED) analysis

All nanoparticles were examined by negative-contrasting TEM. To this end, the material was mounted on nickel grids coated with 1% formvar in dichloroethane. A Libra 120 electron microscope (Carl Zeiss, Germany) operating at 80 keV was used to take photomicrographs. The size, shape, and relative number of electron-dense nanoparticles were evaluated from the TEM images. For SAED analysis, samples were mounted on nickel grids coated with 1% formvar in dichloroethane. The analysis was conducted with a Libra 120 TEM instrument operated at 120 keV (camera length, 360 mm). Both analyses were done at the Simbioz Center for the Collective Use of Research Equipment in the Field of Physical–Chemical Biology and Nanobiotechnology at the Russian Academy of Sciences’ Institute of Biochemistry and Physiology of Plants and Microorganisms.

### UV spectroscopy

This was used to examine the optical properties of the nanoparticles. Absorption spectra were measured with a Specord 250 UV–vis spectrophotometer (Analytik Jena, Germany) at 190–1100 nm.

### Zeta-potential measurements

The *ζ*-potential of the nanoparticles was measured with a Zetasizer Nano ZS instrument (Malvern, UK).

### Assay for cytotoxicity of Au and Ag nanoparticles

The cytotoxicity of the Au and Ag nanoparticles was evaluated with a standard MTT [3-(4,5-dimethylthiazol-2-yl)-2,5-diphenyltetrazolium bromide] assay (Niks & Otto, 1990), with minor modifications. HeLa (human breast cancer) cells and Vero (kidney epithelial cells extracted from an African green monkey) cells were obtained from the Institute of Cytology, Russian Academy of Sciences, and were grown in complete Dulbecco’s modified Eagle’s medium (DMEM; Biolot, Russia) containing 10% fetal bovine serum (Biolot), 100 µg/mL of penicillin, and 100 µg/mL of streptomycin (both from Sigma-Aldrich, USA). Au and Ag nanoparticles were formed by 30-min incubation of HAuCl_4_ or AgNO_3_ with *L. edodes* extracellular extracts at 25 °C. The suspension was centrifuged for 15 min at 15,000 g and the pellet was resuspended in DMEM to a final nanoparticle concentration of 100 µg/mL.

The cells were grown in 25 cm^2^ tissue culture flasks in a water-jacketed incubator at 37 °C in a humidified atmosphere of 5% CO_2_. Twenty-four hours before the assay, the cells were seeded in 96-well plates (about 20,000 cells per well). Then, the cell medium was refreshed and double dilutions of the nanoparticle solutions (1:10, v/v) were added to each well (starting concentration, 100 µg/mL). The controls were cells grown without nanoparticles. After incubation for 24 and 48 h, the cell medium was removed, and 100 µL of 5 mg/mL of MTT (Sigma-Aldrich) in 0.01 M phosphate-buffered saline (PBS, pH 7.4; Biolot) was added to each well. The plates were incubated at 37 °C for 1 h in the dark in a humidified atmosphere of 5% CO_2_. After that, the supernatant liquid was decanted, 100 µL of dimethyl sulfoxide (C_2_H_6_OS; Reachim, Russia) was added to each well, and the plates were incubated at 37 °C for 15 min to resuspend the precipitate of formazan. Absorbance was read at 490 nm with a Spark 10M microplate spectrophotometer (Tecan, Austria). The respiratory activity of the cells grown in a particle-free medium was taken as 100%. Each experiment was at least triplicated, and the standard deviation was used to estimate the error. Before and after the nanoparticles were added, the cell monolayer was directly observed in the bright-field mode by using a Leica DMI3000 B inverted microscope equipped with a Leica 420 D CCD camera (Leica Microsystems, Germany; provided by the Simbioz Center for the Collective Use of Research Equipment in the Field of Physical–Chemical Biology and Nanobiotechnology at the Russian Academy of Sciences’ Institute of Biochemistry and Physiology of Plants and Microorganisms).

### Statistics

There were five independent experiments, each having no less than five replications. Data were processed with Excel software (Microsoft Corp., Redmond, WA, USA).

## Results

### Characterization of Au and Ag nanoparticles reduced with intra- and extracellular extracts

**Figure 1:**
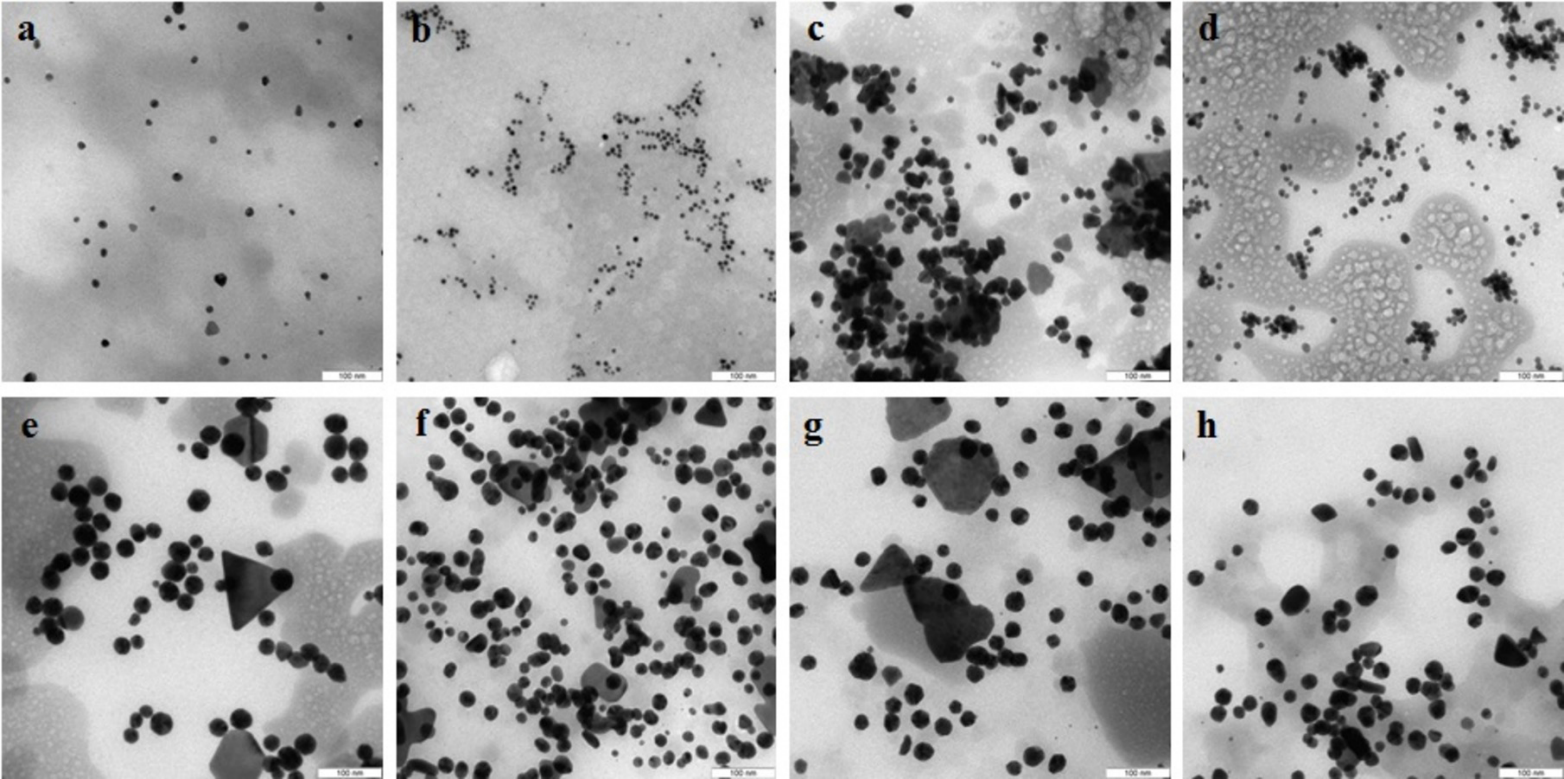
TEM of Au nanoparticles. Nanoparticles produced from HAuCl_4_ with extracellular (A–D) and intracellular (E–H) extracts of *L. edodes* (A, E), *P. ostreatus* (B, F), *G. lucidum* (C, G), and *G. frondosa* (D, H). Bar marker –100 nm.

TEM showed that depending on the extract type and on the compound reduced, the nanoparticles made by different fungi differed widely in shapes and sizes. The reduction of HAuCl_4_ with extracellular extracts yielded sufficiently homogeneous, mostly spherical particles of 2 to 20 nm diameter (Figs. 1A–1D). With *G. lucidum* and *G. frondosa*, the particles were larger, less homogeneous, and often stuck together in aggregates (Figs. 1C and 1D). The Au particles obtained with intracellular extracts were sevenfold larger than those obtained with extracellular extracts. Some particles, of hexagonal, tetragonal, and triangular shape, were even larger (50–100 nm; Figs. 1E–1H). The synthesized Au nanoparticles had a plasmon resonance peak at 550 nm. The *ζ*-potentials of the colloidal Au ranged from −15 to −27 mV. The colloids of Au nanoparticles were stable (except that of *G. lucidum*), and the particles did not aggregate or flocculate for 90 days at 4 °C.

**Figure 2:**
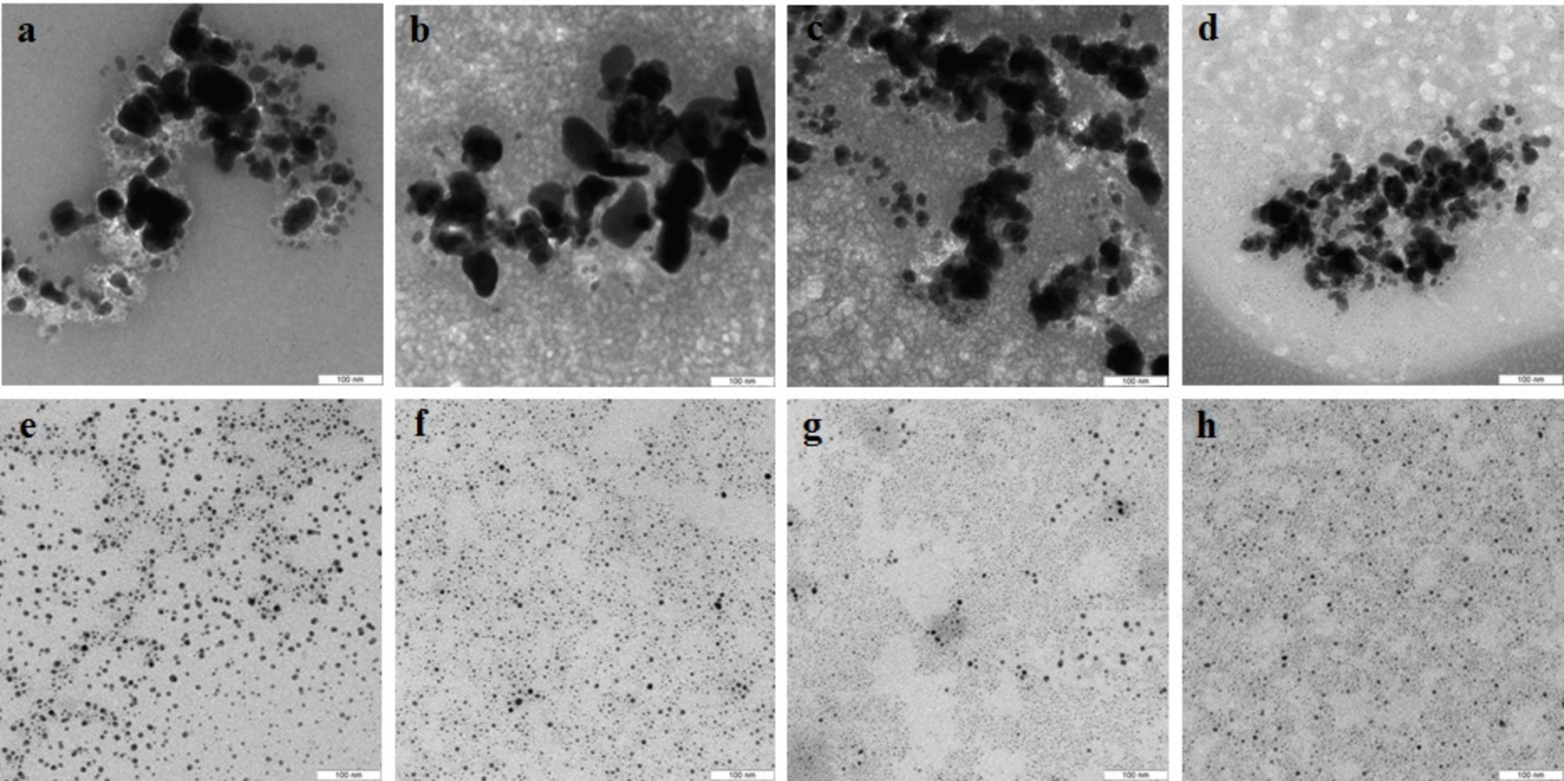
TEM of Ag nanoparticles. Nanoparticles produced from AgNO_3_ with extracellular (A–D) and intracellular (E–H) extracts of *L. edodes* (A, E), *P. ostreatus* (B, F), *G. lucidum* (C, G), and *G. frondosa* (D, H). Bar marker –100 nm.

In the case of reduction of AgNO_3_ with extracellular extracts, the particles formed were large, irregular, and aggregated (Figs. 2A–2D). The *ζ*-potentials of the colloidal Ag solutions ranged from –9 to –12 mV. With mycelial extracts, very small homogeneous particles formed that had diameters of 1 to 5 nm (Figs. 2E–2H). The *ζ*-potential of these particles was 20 mV. The colloids were stable, and the particles did not aggregate or flocculate for 30 days at 4 °C. UV spectroscopy of a suspension of Ag nanoparticles in the culture liquid showed a major absorption peak at 370 nm.

### Effect of phenol oxidase activity of extracts on biodegradation of metals and metalloid compounds

**Figure 3:**
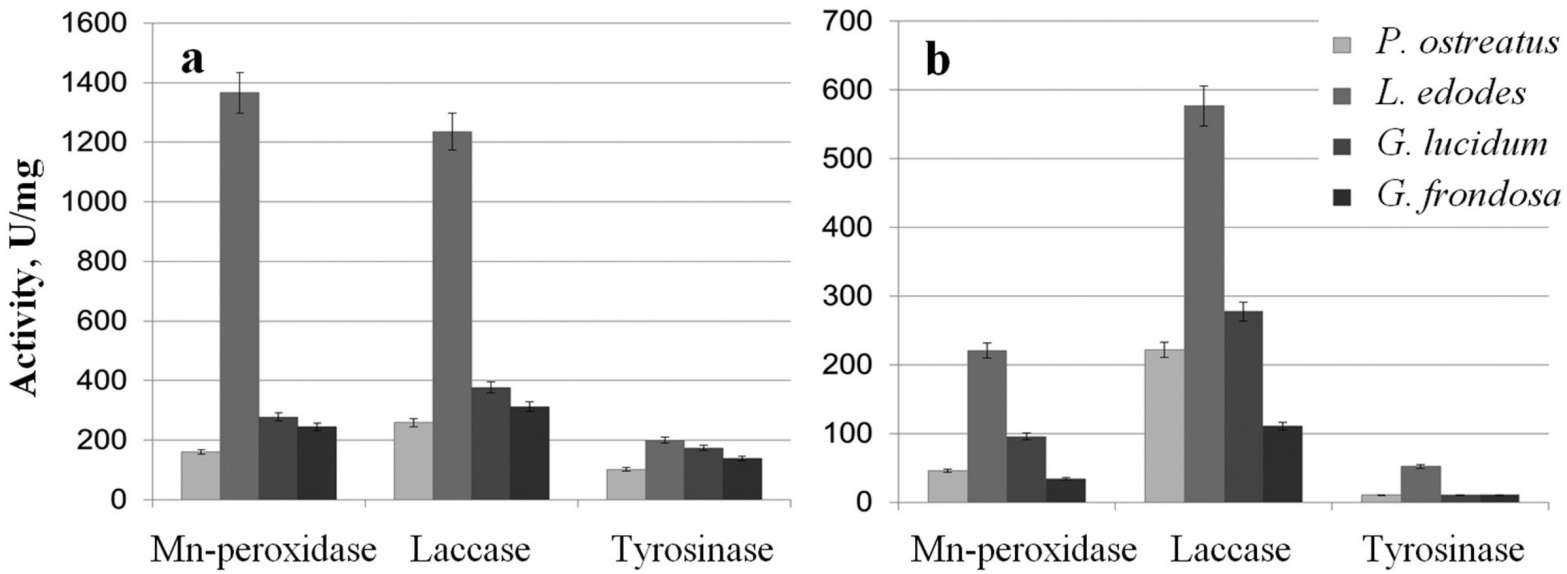
Phenol oxidases activites. Mn-peroxidase, laccase, and tyrosinase activities in extracellular (A) and intracellular (B) extracts of *P. ostreatus*, *L. edodes*, *G. lucidum*, and *G. frondosa.*

The extracts were examined for the activity of Mn-peroxidases, laccases, and tyrosinases. The activity of *L. edodes* Mn-peroxidases in the extracellular extracts was fivefold higher than that in the extracts of *G. lucidum* and was sevenfold higher than that in *P. ostreatus* (Fig. 3A). Total laccase activity in *L. edodes* was 4.5-fold greater than that in *P. ostreatus* and was 3–3.5-fold greater than that in *G. lucidum* and in *G. frondosa*. Tyrosinase activity in submerged fungal cultures was relatively low, but it was somewhat higher in *L. edodes* than in the other fungi. The intracellular extracts of the submerged cultures gave a similar picture, and the most active enzymes were those of *L. edodes* (Fig. 3B). The ability to reduce HAuCl_4_ and AgNO_3_ to Au and Ag was directly proportional to enzyme activity. The biofabrication of Se and Si nanoparticles did not depend on phenol oxidase activity.

### Characterization of Se and Si nanoparticles obtained with intra- and extracellular extracts

**Figure 4:**
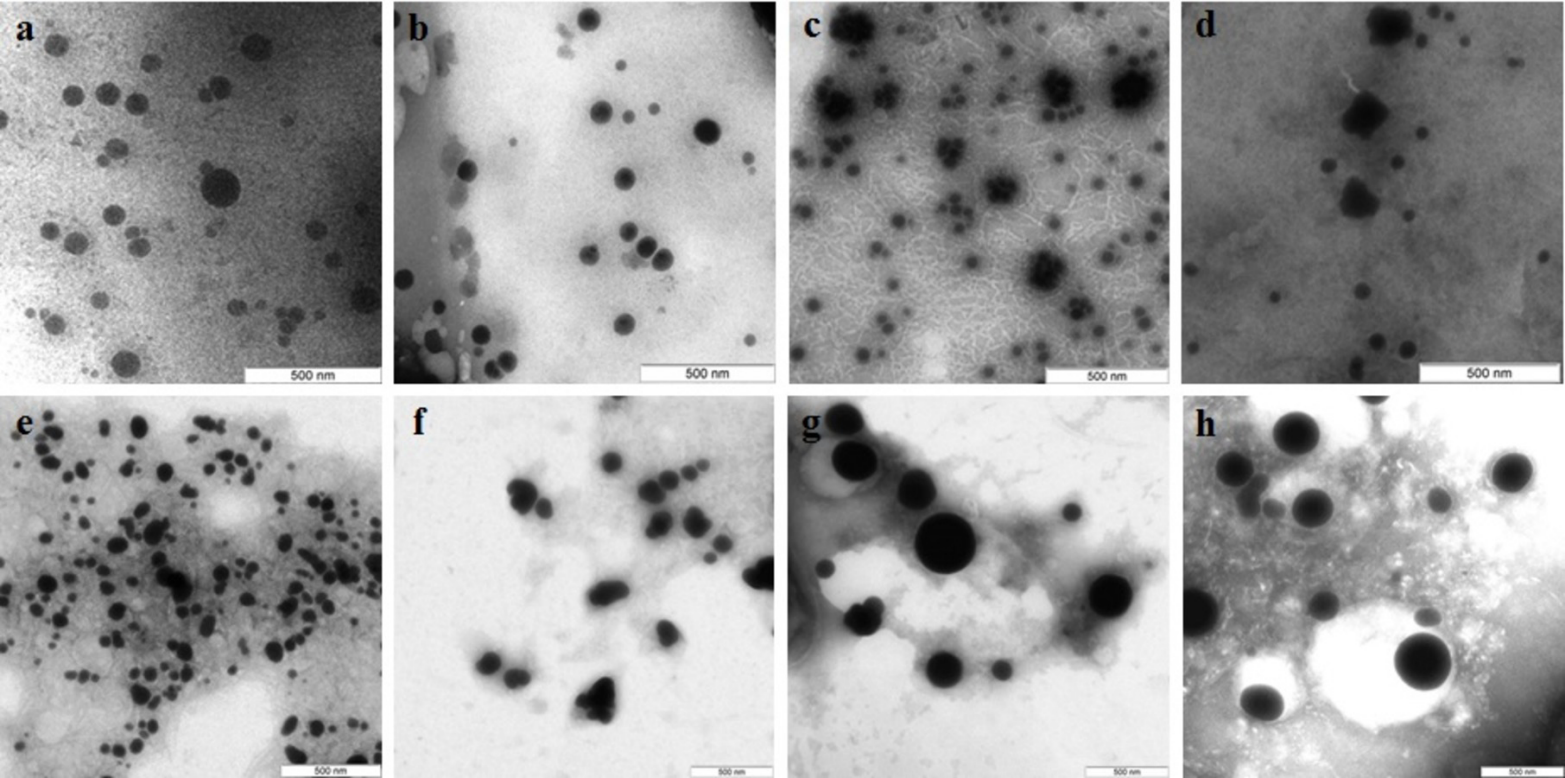
TEM of Se/SeO_2_ nanoparticles. Nanoparticles produced from Na_2_SeO_3_ with extracellular (A–D) and intracellular (E–H) extracts of *L. edodes* (A, E), *P. ostreatus* (B, F), *G. lucidum* (C, G), and *G. frondosa* (D, H). Bar marker –500 nm.

The Se particles formed with the fungal extracts had a more or less regular spherical shape (Fig. 4). In *L. edodes* (Figs. 4A and 4E) and in *P. ostreatus* (Figs. 4B and 4F), the differences between the use of extracellular and intracellular extracts were small and the particle diameter ranged from 50 to 150 nm. Intracellular extracts of *G. lucidum* (Fig. 4G) and *G. frondosa* (Fig. 4H) yielded larger-diameter particles (about 200–300 nm), and their extracellular extracts yielded nanospheres of 20 to 50 nm, often aggregated (Figs. 4C and 4D). The synthesized Se nanoparticles had a plasmon resonance peak at 350 nm. The *ζ*-potentials of the colloidal Se ranged from −12 to −18 mV.

**Figure 5:**
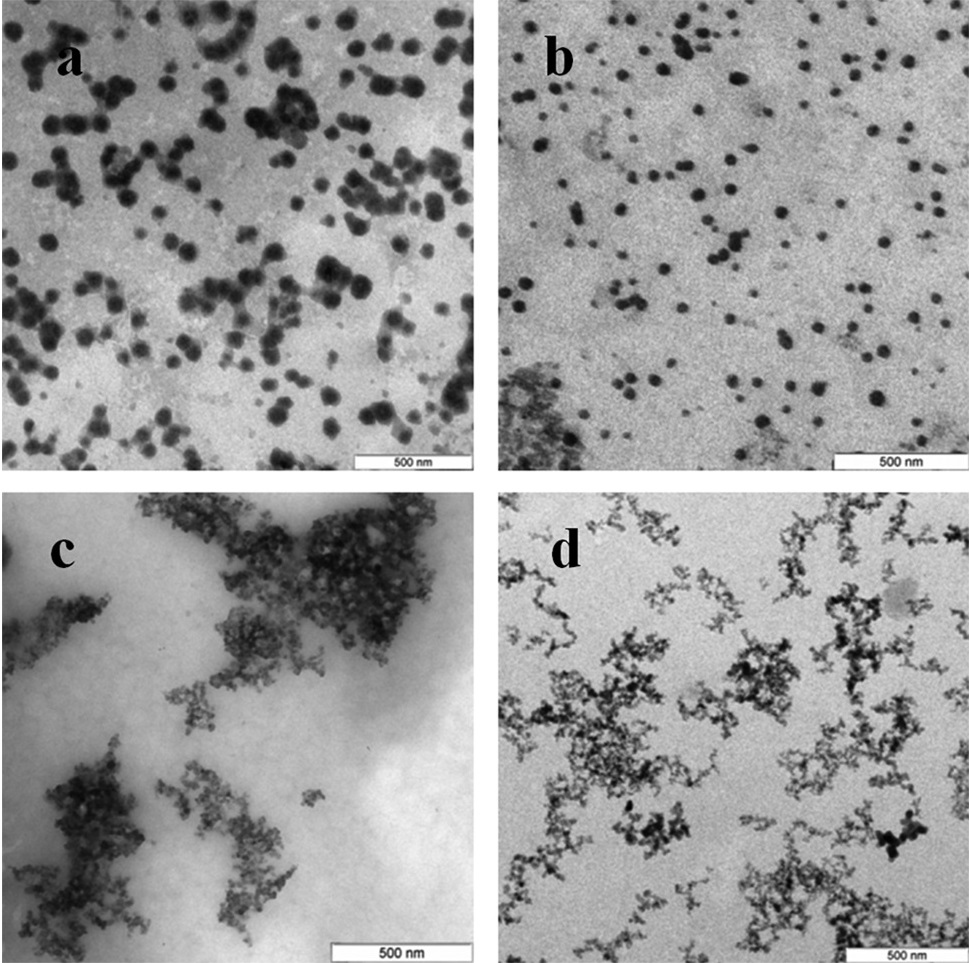
TEM of Si/SiO_2_ nanoparticles. Nanoparticles produced from Na_2_SiO_3_ with extracellular extracts of *L. edodes* (A), *G. lucidum* (B), *P. ostreatus* (C), and *G. frondosa* (D). Bar marker –500 nm.

Si nanoparticles were detected when Na_2_SiO_3_ was incubated with extracellular extracts (Fig. 5). In *L. edodes* and in *G. lucidum*, the particles were larger and did not aggregate (Figs. 5A and 5B), whereas in *P. ostreatus* and in *G. frondosa*, they were very small and stuck together in aggregates (Figs. 5C and 5D). The *ζ*-potentials of the colloidal Si ranged from −10 to −15 mV.

### X-ray fluorescence and X-ray diffraction analysis of nanoparticles

**Figure 6:**
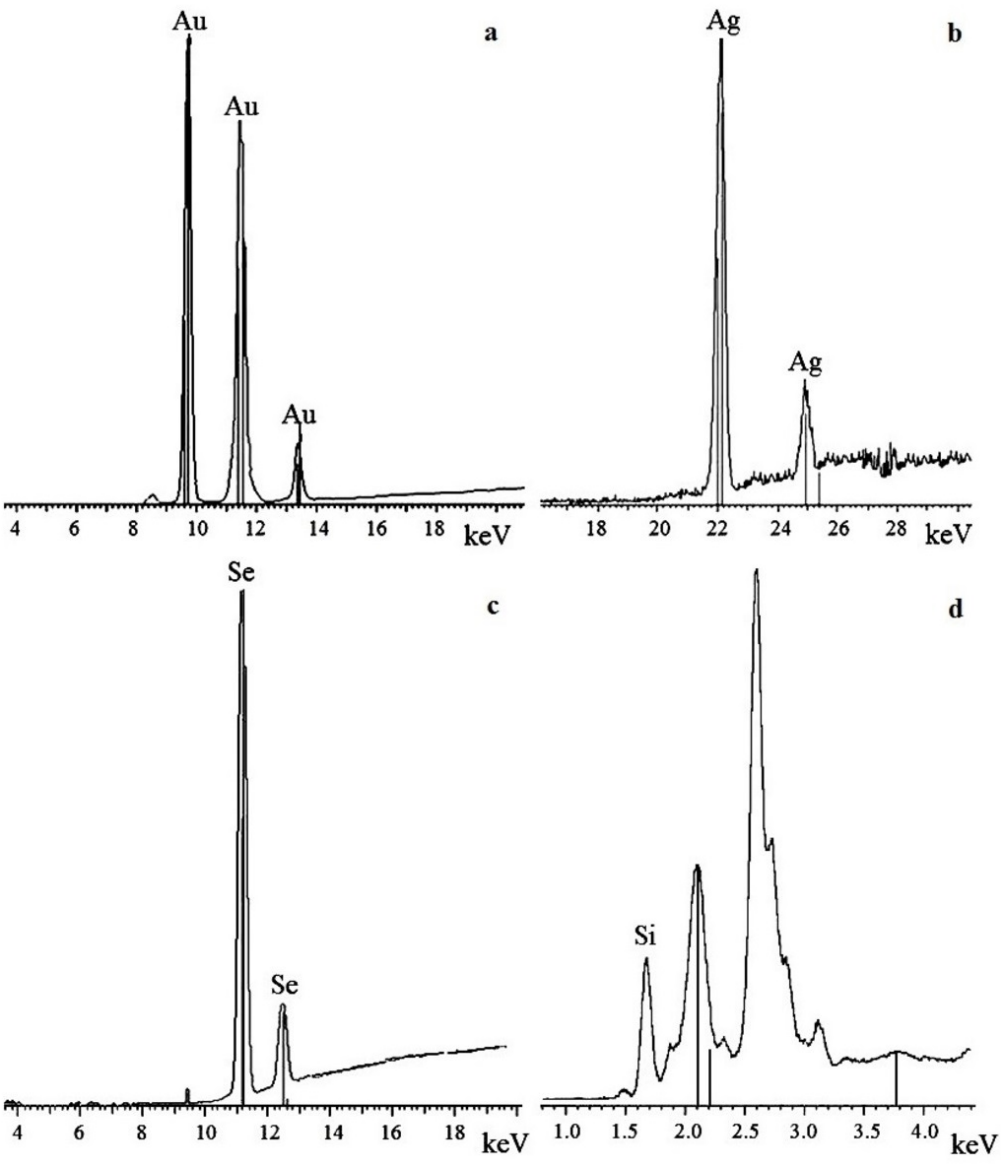
X-ray fluorescence analysis. Au nanoparticles (A), Ag nanoparticles (B), Se nanoparticles (C), and Si nanoparticles (D).

To confirm that the resultant nanoparticles were indeed Au, Ag, Se, and Si, we examined them by X-ray fluorescence. The spectra showed intense emission lines typical of these elements. The presence of Au was determined by the lines at 9.713 (Lα1), 11.443 (Lβ1), 11.585 (Lβ2), 10.308 (Ln), and 13.382 (Ly1) keV (Fig. 6A); the presence of Ag, by the lines at 22 (Lα1) and 25 (Lβ1) keV (Fig. 6B); the presence of Se, by the lines at 11.22 (Lα1) and 12.49 (Lβ1) keV (Fig. 6C); and the presence of Si, by the line at 1.75 (Lα1) keV (Fig. 6D). The elements were detected in all the fungi tested.

**Figure 7:**
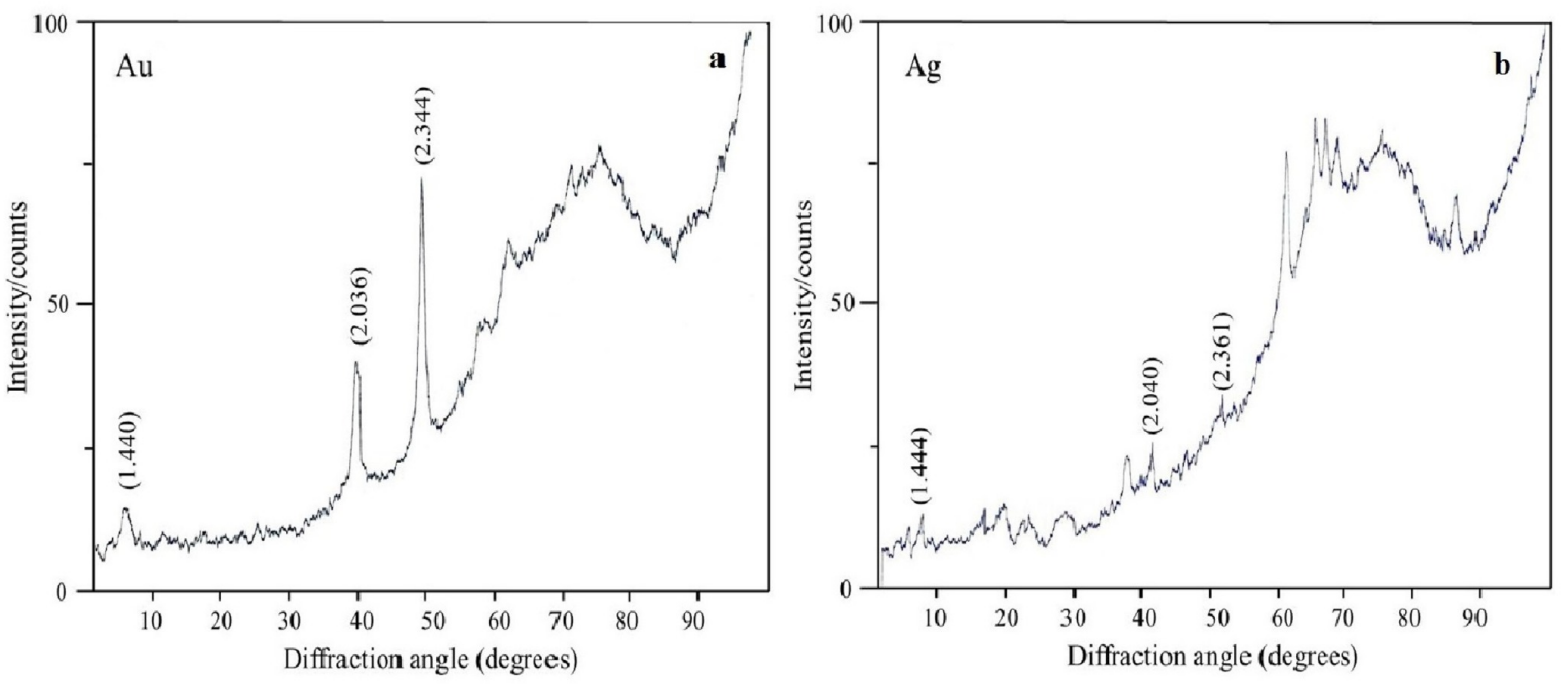
X-ray diffraction analysis. Au nanoparticles (A) and Ag nanoparticles (B) synthesized by *L. edodes* from HAuCl_4_ and AgNO_3_.

The oxidation states of the Au, Ag, Se, and Si in nanoparticle suspensions were examined by X-ray diffraction analysis. The metals were fully reduced to the elementary state, and there were no signals from either HAuCl_4_ or AgNO_3_ (Figs. 7A and 7B). There were four signals belonging to the major reflex of Au (interplanar distances, 1.230, 1.441, 2.030, and 2.350 Å  [4–784]) and four signals corresponding to the major reflex of Ag (interplanar distances, 1.232, 1.446, 2.049, and 2.356 Å [4–783]). Also found were seven minor signals belonging to the major reflex of Se (1.768, 1.993, 2.014, 2.060, 2.171, 2.990, and 3.750 Å  [6–0362]) and two signals from SeO_2_ (2.529 and 4.171 Å [22–1314]). In the samples with Si nanoparticles, there were minor signals belonging to two silicon structures, one with three reflexes (1.630, 1.918, and 3.140 Å [5–565]) and the other with two reflexes (1.957 and 2.280 Å [35–1158]). Also present were five signals that were assigned with high confidence to the major reflex of quartz SiO_2_ (2.228, 2.280, 2.460, 3.351, and 4.230 Å [5-0490]).

### TEM and SAED analysis

**Figure 8:**
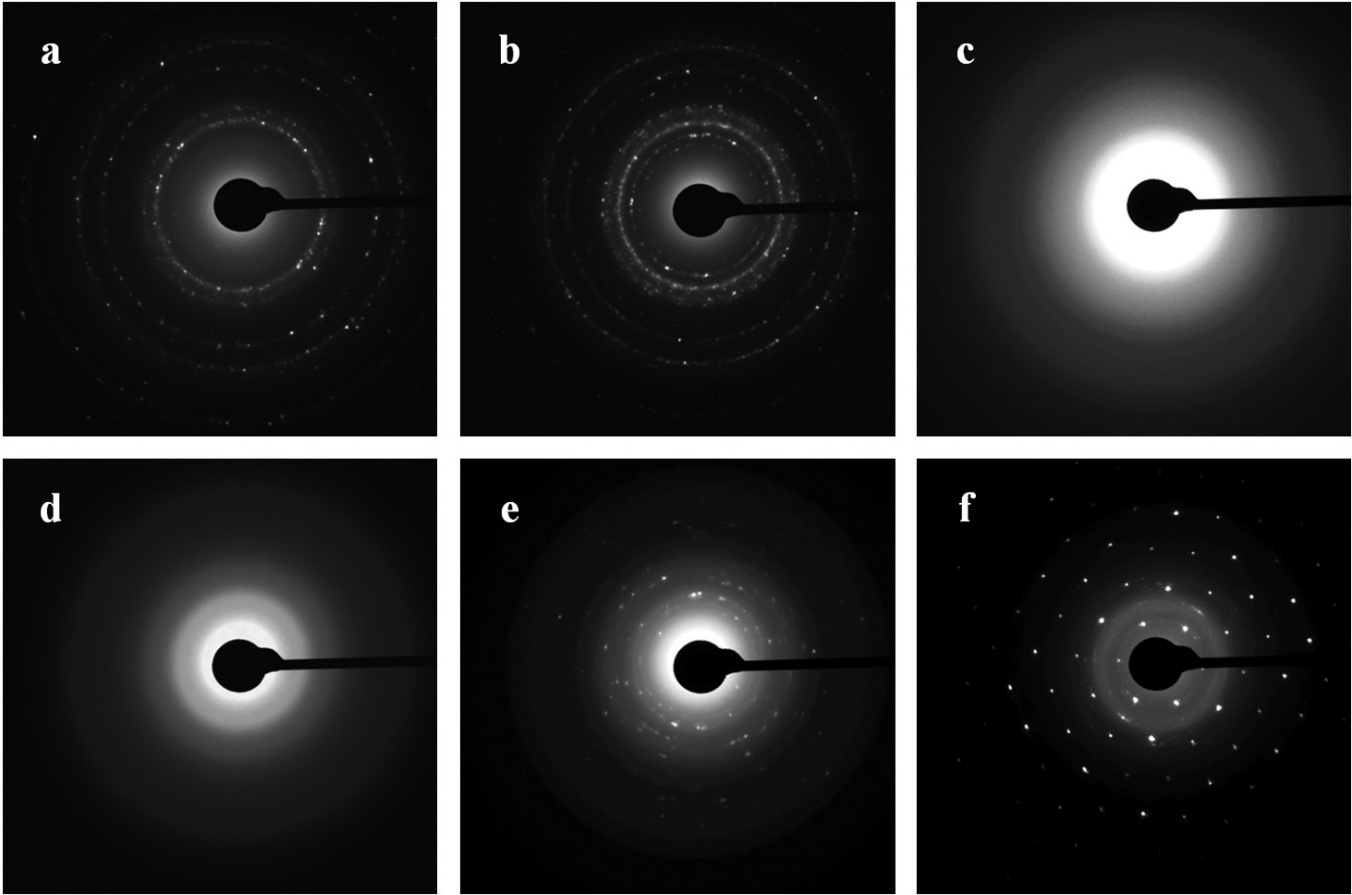
SAED. Patterns presented as Au (A), Ag (B), Se (C), and Si/SiO_2_ (D–F) nanoparticles.

Diffraction patterns of the nanoparticles were obtained by TEM and by SAED analysis. Figure 8 shows the ring diffraction patterns from pure Au (Fig. 8A) and Ag (Fig. 8B) particles with crystalline structure. The Se particles were shown by SAED analysis to be noncrystalline (Fig. 8C). SAED analysis of the Si structures by using individual particle ensembles showed diffused ring patterns typical of amorphous materials, in line with the amorphous nature of the formed Si superstructures (Fig. 8D). There also were diffraction signals from crystalline Si, indicating that Na_2_SiO_3_ transformation was accompanied by the synthesis of Si nanoparticles with crystalline structure (Figs. 8E, 8F).

### Nanoparticle synthesis with intracellular extracts from different stages of fungal development

**Figure 9:**
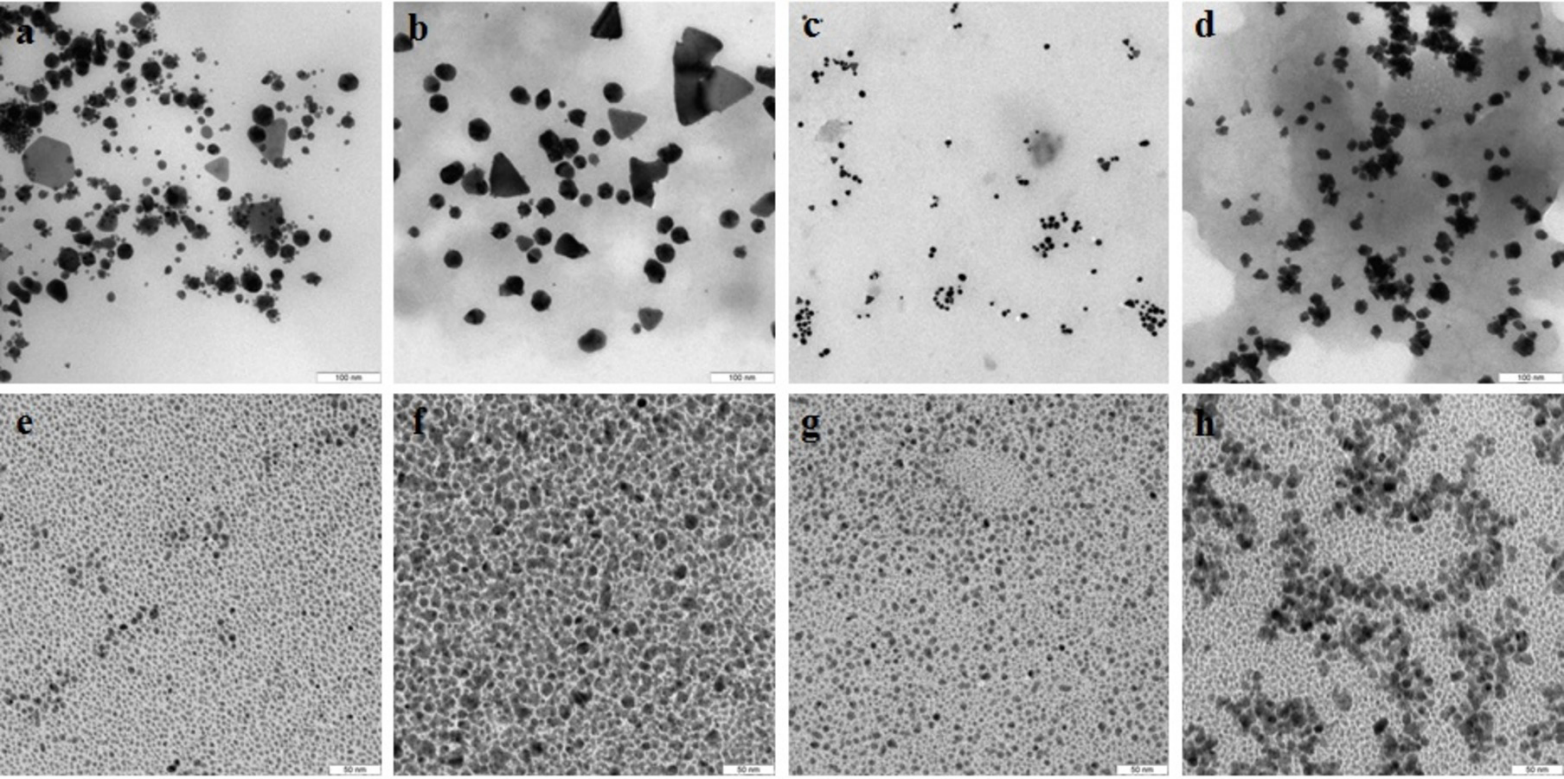
TEM of Au and Ag nanoparticles. Au (A–D) and Ag (E–H) nanoparticles produced from HAuCl_4_ and AgNO_3_, respectively, at different stages of *L. edodes* morphogenesis: nonpigmented mycelium (A, E), pigmented mycelium (B, F) brown mycelial mat (C, G), and primordia (D, H). Bar markers –100 nm (Au) and 50 nm (Ag).

With extracts of nonpigmented mycelia, the synthesized Au particles had an irregular spherical shape and a size of 2–50 nm. Also formed were a small number of nanotriangles (50–80 nm) and hexagons (20–90 nm) (Fig. 9A). With pigmented mycelia, 10–40-nm spherical particles and many triangles (up to 100 nm in size) were formed (Fig. 9B). In extracts of brown mycelial mats, we found homogeneous, mostly spherical particles of 5 to 10 nm in size (Fig. 9C). Extracts of primordia reduced HAuCl_4_ to irregular particles about 10–30 nm in diameter, most of which were aggregated (Fig. 9D).

**Figure 10:**
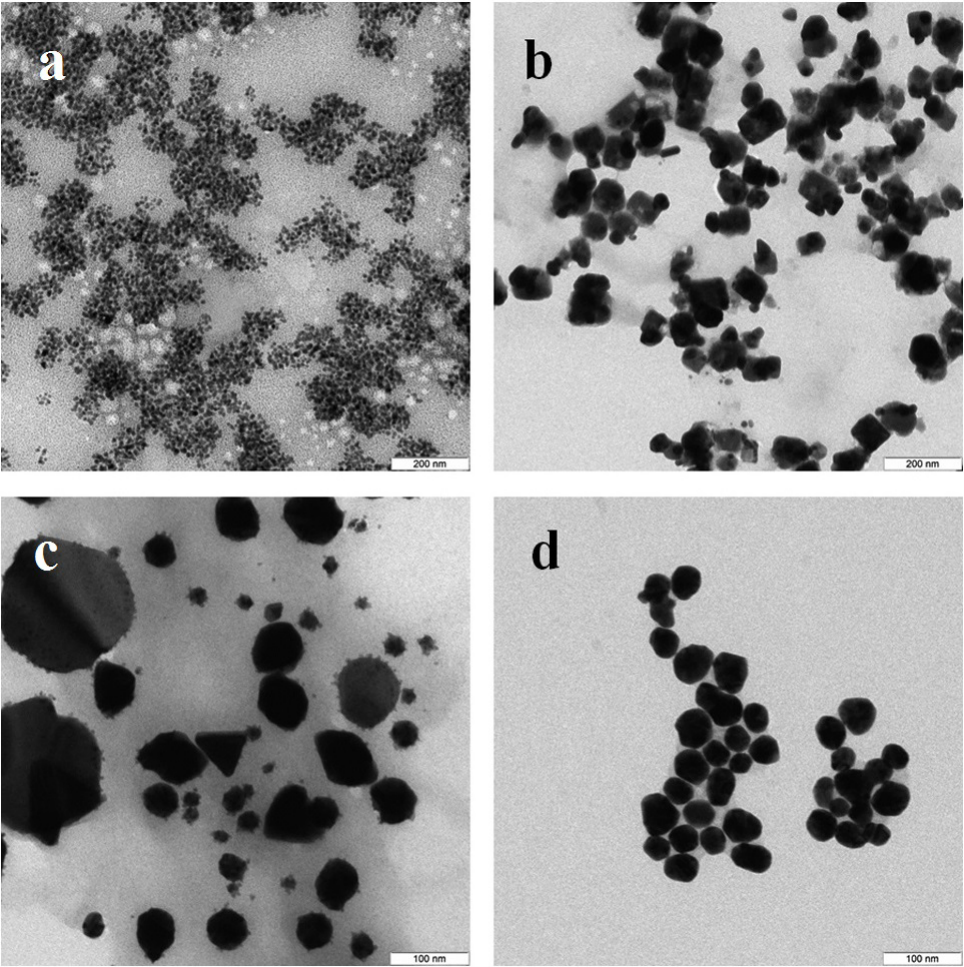
TEM of Ag and Au nanoparticles. TEM of Ag (A, B) and Au (C, D) nanoparticles produced from AgNO_3_ and HAuCl_4_, respectively, by using intracellular extracts from fruiting bodies of *L. edodes* (A, C) and *G. lucidum* (B, D). Bar markers –100 nm (Au) and 200 nm (Ag).

Nanoparticles of Ag were smaller and were homogeneous (Figs. 9E–9H). Extracts of nonpigmented mycelia and brown mycelial mats produced a stable suspension of 1 to 10-nm particles (Figs. 9E and 9G). By contrast, the particles formed with extracts of pigmented mycelia were larger (5–20 nm) (Fig. 9F). A slightly different picture was observed for extracts of *L. edodes* primordia; alongside small, homogeneous nanoparticles (2–5 nm), there were many 10 to 20-nm particles stuck together in aggregates (Fig. 9H). With extracts of *L. edodes* fruiting bodies, there were many small spherical nanoparticles of 10 to 15 nm (Fig. 10A). With *G. lucidum*, the particles were large (50–100 nm) and mostly cubic and rectangular (Fig. 10B). Incubation of HAuCl_4_ with fruiting body extracts of *L. edodes* yielded inhomogeneous particles, among them many large irregular spheres of up to 170 nm, some triangles of 60–90 nm, and some irregular spheres of 15 to 50 nm (Fig. 10C). Extracts from *G. lucidum* synthesized mostly spheres of 30–50 nm (Fig. 10D).

### Assay for cytotoxicity of nanoparticles

**Figure 11:**
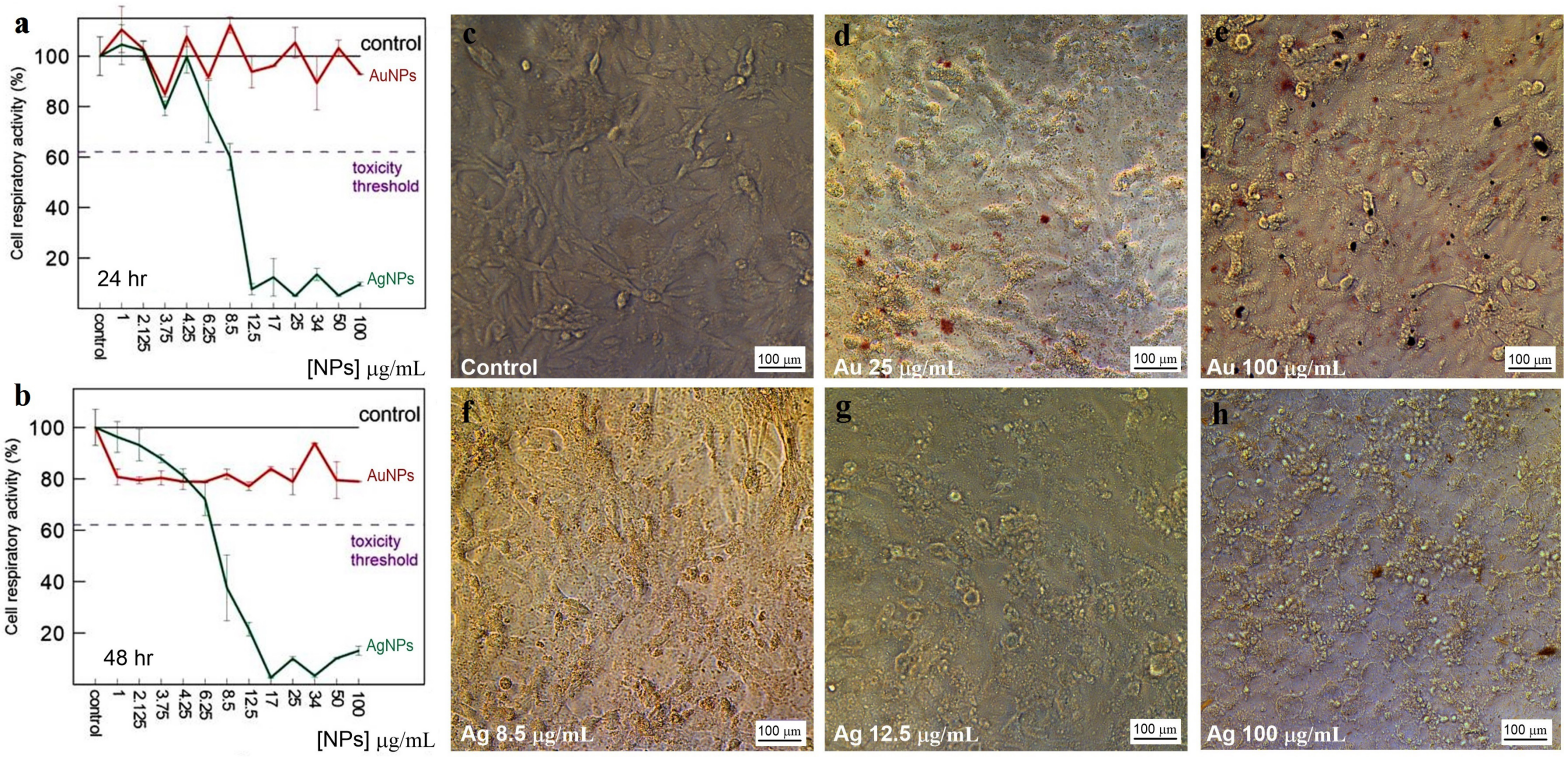
Cytotoxicity of Ag and Au nanoparticles on Vero cells. Respiratory activity of Vero cells analyzed by MTT assay, incubated for 24 (A) and 48 h (B) with Ag and Au nanoparticles produced by using *L. edodes* extracellular extracts, and bright-field microscopy images of pure Vero cells as control (C) and cells incubated for 24 h with nanoparticles in varying concentrations (D–H). Bar markers –100 µm.

**Figure 12:**
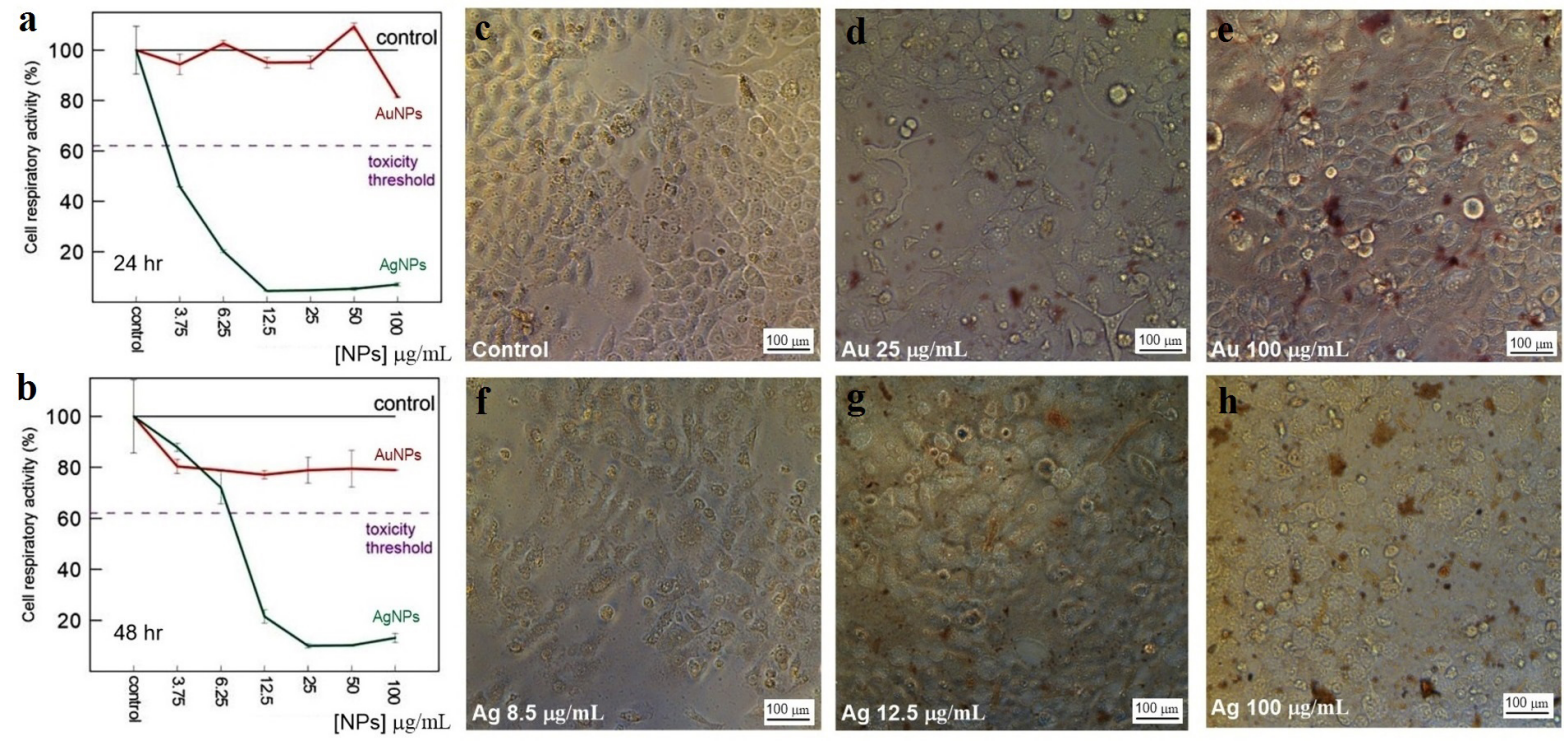
Cytotoxicity of Ag and Au nanoparticles on HeLa cells. Respiratory activity of HeLa cells analyzed by MTT assay, incubated for 24 (A) and 48 h (B) with Ag and Au nanoparticles produced by using *L. edodes* extracellular extracts, and bright-field microscopy images of pure HeLa cells as control (C) and cells incubated for 24 h with nanoparticles in varying concentrations (D–H). Bar markers –100 µm.

The toxicity of Au and Ag nanoparticles to HeLa and Vero cells was evaluated with a standard MTT assay. The average decrease in cell respiratory activity of both Vero (Figs. 11A, 11B) and HeLa (Figs. 12A, 12B) cells, as compared to the control, was about 20% within 24 and 48 h with all Au particle concentrations tested (1–100 µg/mL). This indicated that the cell toxicity had a non-dose–response character. Ag nanoparticles were strongly toxic at as low as 3.75 µg/mL (HeLa) and 8.5 µg/mL (Vero) after being incubated with the cells for 24 and 48 h (Figs. 11 and 12). The presence of unattached dead cells indicated partly or totally disrupted cell monolayer (Figs. 11C–11H and Figs. 12C–12H).

## Discussion

When HAuCl_4_, AgNO_3_, and Na_2_SeO_3_ were incubated with culture liquid filtrates (extracellular extracts) and with intracellular extracts from the fungi used, the solutions acquired characteristic red–lilac, ginger brown, and orange–red colorations. These colorations indicated the synthesis and accumulation of elementary Au, Ag, and Se, respectively (Philip, 2009; Popescu, Velea & Lőrinczi, 2010; Das, Das & Guha, 2009; Iravani, 2011). The hues and color intensities of the solutions differed depending on the fungal species and, particularly, on the extract type. These differences indicated that differently shaped and sized particles had formed. When the extracts were incubated with Na_2_SiO_3_, no coloration was observed, because a suspension of Si nanoparticles is colorless. Our finding that extra- and intracellular extracts are capable of reduction of metal and metalloid ions, including bioformation of Au, Ag, Se, and Si nanoparticles, agrees with our earlier results obtained with basidiomycetes grown submerged with HAuCl_4_, AgNO_3_, Na_2_SeO_3_, and Na_2_SiO_3_ (Vetchinkina et al., 2013a; Vetchinkina et al., 2013b; Vetchinkina et al., 2014; Vetchinkina et al., 2016; Vetchinkina et al., 2017). In those studies, we have shown in vivo that living fungal cultures can produce nanoparticles and can deposit them in large quantities on the surface of and inside the mycelial hyphae, as well as extracellularly.

Our previous and current findings show that the reduction of ions and the subsequent synthesis of Au, Ag, Se, and Si nanoparticles are directly linked to fungal metabolism. The extracellular extracts, containing proteins and enzymes, and the intracellular extracts equally effectively reduce the compounds to the elementary state with the formation of nanoparticles. Microbial reduction of metal compounds may implicate microbial enzymes (Durán, Silveira & Durán, 2015; Faramarzi & Forootanfara, 2011; Sanghi, Verma & Puri, 2011; El-Batal et al., 2015). The potent oxidation and reduction system of the xylotrophic fungi is formed by the phenol oxidases—Mn-peroxidases, laccases, and tyrosinases. We, therefore, logically assumed that these enzymes would play a part in the synthesis of nanoparticles with extracts from the fungi used in this work. The intra- and extracellular extracts were examined for the activity of Mn-peroxidases, laccases, and tyrosinases, because these enzymes are present in fungi both extra- and intracellularly (Vetchinkina, Pozdnyakova & Nikitina, 2008). Phenol oxidases were most active in a submerged culture of *L. edodes*. By contrast, in submerged cultures of *P. ostreatus*, *G. lucidum*, and *G. frondosa*, phenol oxidase activity was severalfold lower, depending on the fungal species and on the specific enzyme. This difference in activity can be explained by the use of different growth conditions. When *G. lucidum* was grown submerged on the mineral medium lacking the wood substrate, its phenol oxidase activity was relatively low. This indicates that these growth conditions were unusual for this fungus, which has a strong phenol oxidase complex. On the contrary, *L. edodes* was better adapted to growth under these conditions. In the *L. edodes* extracts, with higher phenol oxidase activity, nanoparticles were reduced and synthesized faster. As early as several minutes into incubation, the solutions acquired intense red–lilac (Au) and ginger (Ag) colorations. Reduction with extracts from the other fungi was slower, taking several hours to complete. It may be that other enzymes and proteins present in the extracts also take part in bioreduction and in particle synthesis and that these reactions are affected by all reducing agents together. But several studies have argued for enzymatic synthesis with phenol oxidases as the most probable mechanism. For example, the reduction of auric compounds involves a laccase from the ascomycete *Paraconiothyrium variabile* (Faramarzi & Forootanfara, 2011) and laccases and ligninases from the basidiomycete *Phanerochaete chrysosporium* (Sanghi, Verma & Puri, 2011). Ag nanoparticles associated with Ag chloride nanoparticles were obtained from aqueous Ag nitrate with a semipurified laccase from *Trametes versicolor* (Durán et al., 2014). Our earlier work with intracellular laccases, tyrosinases, and Mn-peroxidases from a submerged culture of *L. edodes* suggests, too, that basidiomycetes use these enzymes to produce metal nanoparticles. After incubation with HAuCl_4_ and AgNO_3_, aqueous solutions of homogeneous enzymes reduced Au and Ag to the elementary state within several minutes, and stable colloids of variously shaped and sized nanoparticles were formed (Vetchinkina et al., 2017). On the basis of the ability of phenol oxidases to reduce noble metals, we proposed possible schemes for these reactions (Kupryashina, Vetchinkina & Nikitina, 2017; Vetchinkina et al., 2017).

In this study, the bioformation of Se and Si nanoparticles from Na_2_SeO_3_ and Na_2_SiO_3_ did not depend on phenol oxidase activity. It is possible that other enzymes, such as NADH-dependent nitrate and nitrite reductases, are implicated in the synthesis of Se and Si nanoparticles. Selenium exists in several forms of condensed state: crystalline (trigonal; hexagonal; α-, β-, and *γ*-monoclinic; rhombohedric; orthorhombic; and α- and β-cubic Se), ultradispersive amorphous Se (red, brown, and black), vitreous Se, and liquid forms obtained as a result of melting of crystalline modifications (Minaev, Timoshenkova & Kalugina, 2005). The red coloration is characteristic for both amorphous Se and monoclinic crystalline Se (Johnson et al., 1999). We detected changes in the color of fungal extracts in the presence of Na_2_SeO_3_ to various hues of pink and red, which indicated the reduction of Se^IV^ to Se^0^ and the accumulation of red Se. The Se particles were shown by SAED analysis to be noncrystalline. In addition, X-ray diffraction analysis detected seven minor signals belonging to the major reflex of crystalline Se. Green synthesis with bacteria, fungi, and other organisms affords nanoparticles of both red amorphous Se (Dhanjal & Cameotra, 2010; Sarkar et al., 2011; Mollania, Tayebee & Narenji-Sani, 2016) and red crystalline Se (Zhang et al., 2011; Srivastava & Mukhopadhyay, 2013).

On the basis of literature data, we speculate than the nanoparticles from the incubation of culture liquid filtrates with Na_2_SiO_3_ were most probably composed of SiO_2_. The biosynthesis of SiO_2_ nanoparticles has been best studied in diatom algae, whose exoskeleton consists of SiO_2_ nanoparticles (50–100 nm) and has a highly ordered structure (Dhillon et al., 2012). By contrast, fungal synthesis of such nanoparticles has received very little attention. Specifically, Bansal et al. (2005a) reported that *Fusarium oxysporum* secretes proteins that extracellularly hydrolyze an aqueous anionic complex of SiF${}_{6}^{2-}$ at room temperature, with the formation of SiO_2_ nanoparticles. *F. oxysporum* may also be used in the bleaching of silica nanoparticles from sand grains (Bansal et al., 2005b). Bioleaching of waste material such as fly ash by *F. oxysporum* resulted in the extracellular production of highly crystalline, highly stable, protein capped, fluorescent, water-soluble silica nanoparticles of quasi-spherical morphology (Khan et al., 2014). The bioleaching of borosilicate glass by the fungus *Humicola* sp. enabled synthesis of nearly monodispersed ultrafine 5 nm silicate nanoparticles (Kulkarni et al., 2008). The synthesis of silicon/silica nanoparticle composites by the bacterium *Actinobacter* sp. occurs when the bacterium is exposed to the K_2_SiF_6_ precursor (Singh et al., 2008).

In *L. edodes*, the compositions of the phenol oxidase complex (intracellular Mn-peroxidases, laccases, and tyrosinases) and the activities of these enzymes differ at different stages of ontogenesis (Vetchinkina et al., 2015). In this study, the color hues and the stabilities of the suspensions differed depending on which morphological structure was implicated in the reduction. Phenol oxidase activity was highest in brown mycelial mats. In their extracts, we observed the most intense synthesis of many small homogeneous nanoparticles, with the formation of stable colloidal solutions of Au and Ag. Metal nanoparticles are often synthesized with extracts from fungal fruiting bodies (Narasimha et al., 2011; Dhanasekaran et al., 2013; El-Sonbaty, 2013; Manzoor-ul haq et al., 2014; Sudhakar et al., 2014). This approach has several strong points: fruiting bodies can be purchased, they produce copious biomass for the preparation of extracts, and the method itself is not time-consuming or costly. In this work, extracts from the fruiting bodies of different fungi produced different Au and Ag nanoparticles. The particles were formed relatively fast: the solution color changed as early as within 1 to 2 h. Several studies using extracts of *Agaricus bisporus* fruiting bodies have reported longer synthesis periods—12 to 72 h (Dhanasekaran et al., 2013). In addition, the particle size had a fairly large scatter—8 to 50 nm (Narasimha et al., 2011; Dhanasekaran et al., 2013; El-Sonbaty, 2013; Manzoor-ul haq et al., 2014; Sudhakar et al., 2014). A comparison of the results shows that the Au and Ag nanoparticles obtained with mycelial mat extracts of *L. edodes* are more homogeneous, have a smaller size, and are spherical; therefore, they are better suited for biotechnological applications. According to the literature data, the anticancer and antimicrobial effect of Ag nanoparticles depends strongly on their size, shape, and concentration. Particles with smaller diameters are better able to penetrate the membranes and damage bacterial cells (Kim et al., 2012; Franci et al., 2015). The characteristics of Au nanoparticles also depend highly on their size and shape. Au nanoparticles of small size (10–30 nm), which are also spherical and homogeneous, can be used in biomedicine, for example, for the treatment of microbial infections and in the diagnosis and treatment of cancer (Chauhan et al., 2011; Sherwani et al., 2015). Our data indicate that the use of extracts from different stages in fungal morphogenesis makes it possible to prepare Au and Ag nanoparticles of specified size, shape, and degree of aggregation.

## Conclusions

Extra- and intracellular extracts from the edible and medicinal cultivated basidiomycetes *L. edodes, P. ostreatus, G. lucidum,* and *G. frondosa* can be used for the synthesis of Au, Ag, Se, and Si nanoparticles from HAuCl_4_, AgNO_3_, Na_2_SeO_3_, and Na_2_SiO_3_, respectively. The use of these mushrooms for the synthesis of nanoparticles is a convenient and promising method, because these cultures are nontoxic and nonpathogenic. The shape, size, and aggregation of the nanoparticles formed with the culture liquid filtrates and intracellular extracts depend on both the species of fungus and the type of extract used. Therefore, by varying the conditions, it is possible to obtain nanoparticles with the necessary parameters. With cell-free extracts, further separation of nanoparticles from biomass is not required, which simplifies the biotechnological process. The metal bioreduction involves laccases, tyrosinases, and Mn-peroxidases, and the formation of nanoparticles is directly proportional to phenol oxidase activity. When Au and Ag nanoparticles are made with extracts from different morphogenetic stages of *L. edodes* and *G. lucidum*, their size, shape, and degree of aggregation differ between types of extracts and between morphological structures involved. The cytotoxicity of the Au nanoparticles is negligible in a broad concentration range (1–100 µg/mL), whereas the Ag nanoparticles are nontoxic only when used between 1 and 10 µg/mL. Synthesis of nanoparticles with cultivated xylotrophic basidiomycetes holds much promise because it is simple, accessible, and environmentally benign and because it yields nanoparticles of required chemical makeup, shape, and size.

##  Supplemental Information

10.7717/peerj.5237/supp-1 Supplemental Information 1 Raw data

## Additional Information and Declarations

### Data Availability

The following information was supplied regarding data availability: The raw data are provided in  Supplemental Information 1.
