# Supplementary figures and images for: Green synthesis of nanoparticles with extracellular and intracellular extracts of basidiomycetes
Source: PeerJ. 2018 Jul 20;6:e5237. doi: 10.7717/peerj.5237 (PMC6055591; doi:10.7717/peerj.5237)

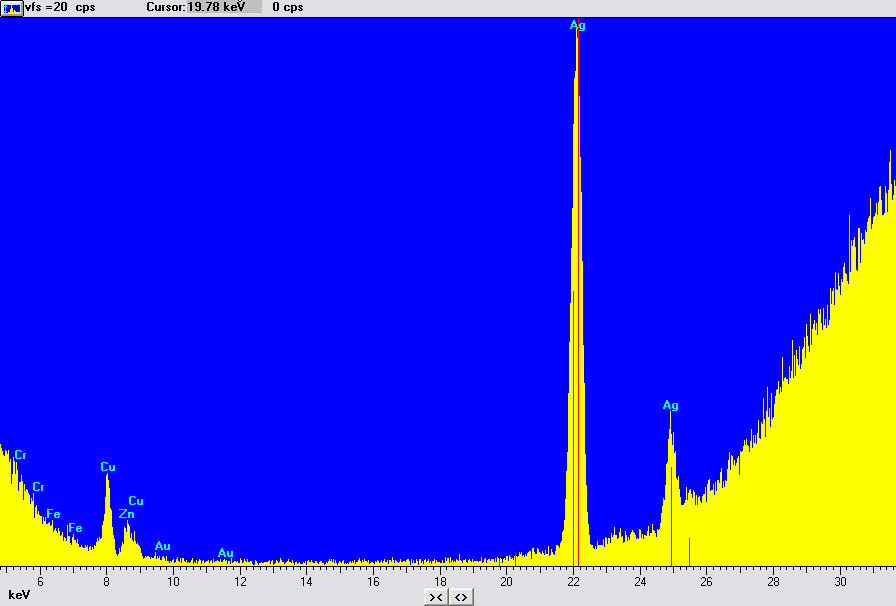

Supplement: Supplemental Information 1 [file peerj-06-5237-s001.zip › RAW/Ag X-ray fl.jpg]

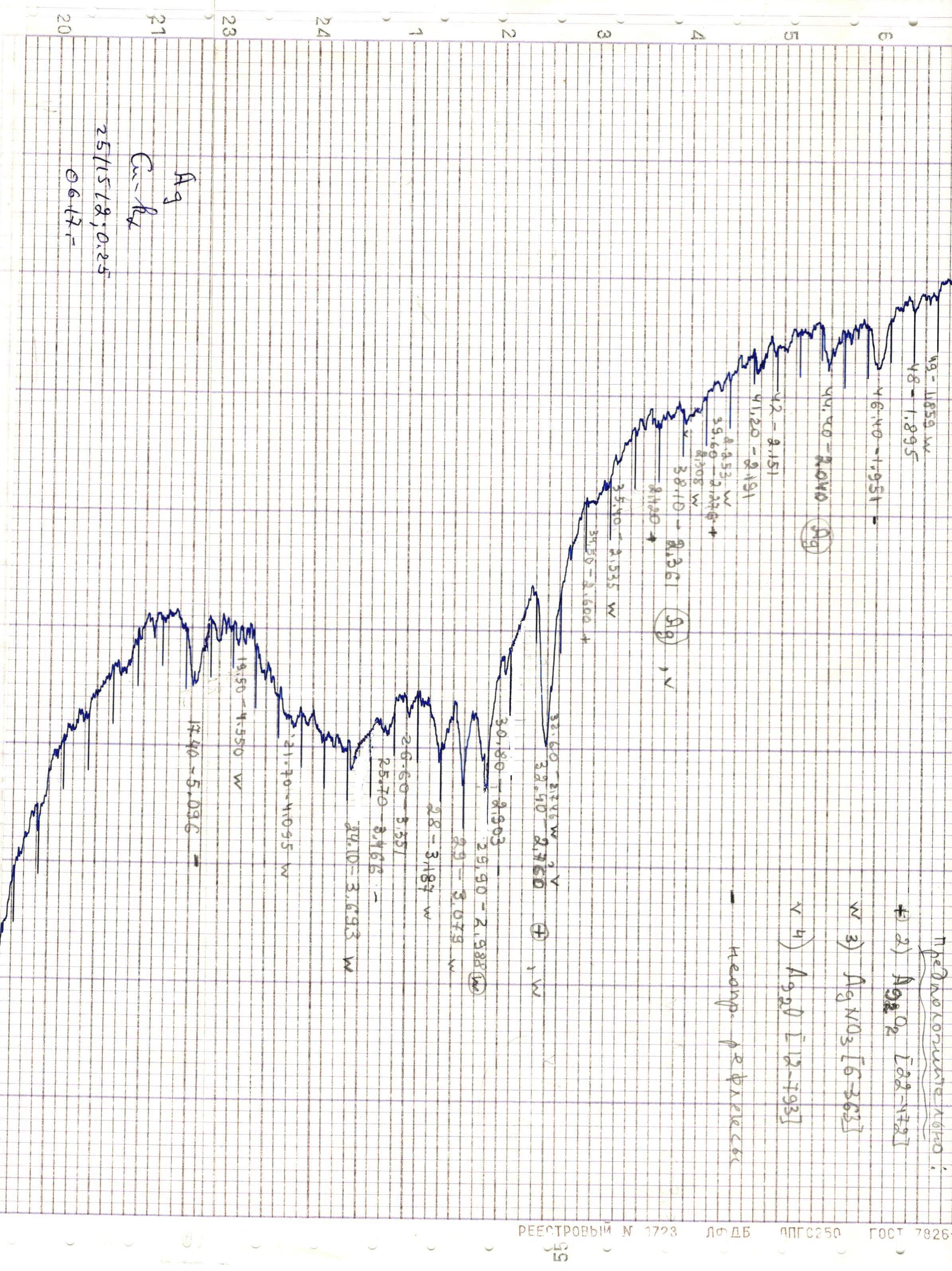

Supplement: Supplemental Information 1 [file peerj-06-5237-s001.zip › RAW/Ag X-ray ph.jpg]

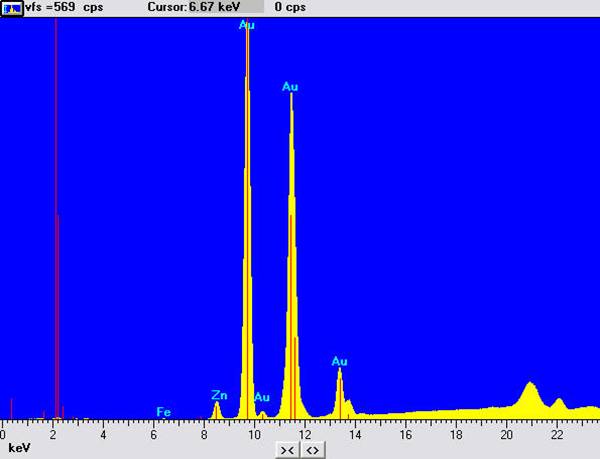

Supplement: Supplemental Information 1 [file peerj-06-5237-s001.zip › RAW/Au X-ray fl.jpg]

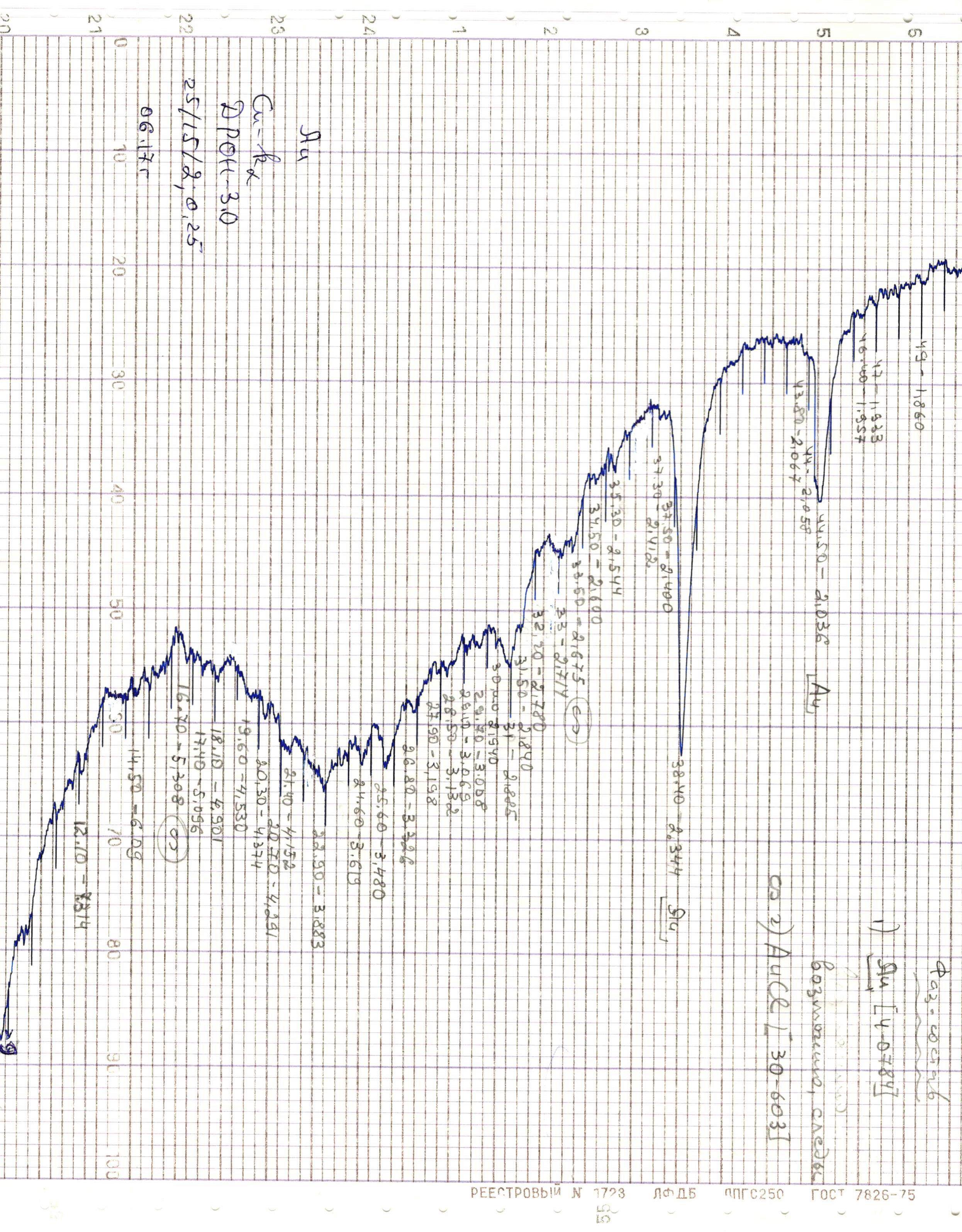

Supplement: Supplemental Information 1 [file peerj-06-5237-s001.zip › RAW/Au X-ray ph.jpg]

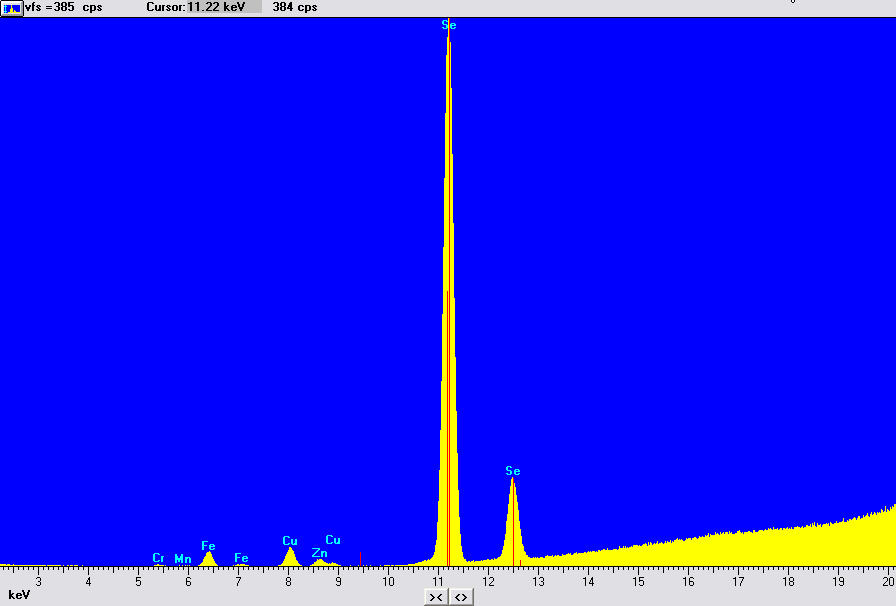

Supplement: Supplemental Information 1 [file peerj-06-5237-s001.zip › RAW/Se X-ray fl.jpg]

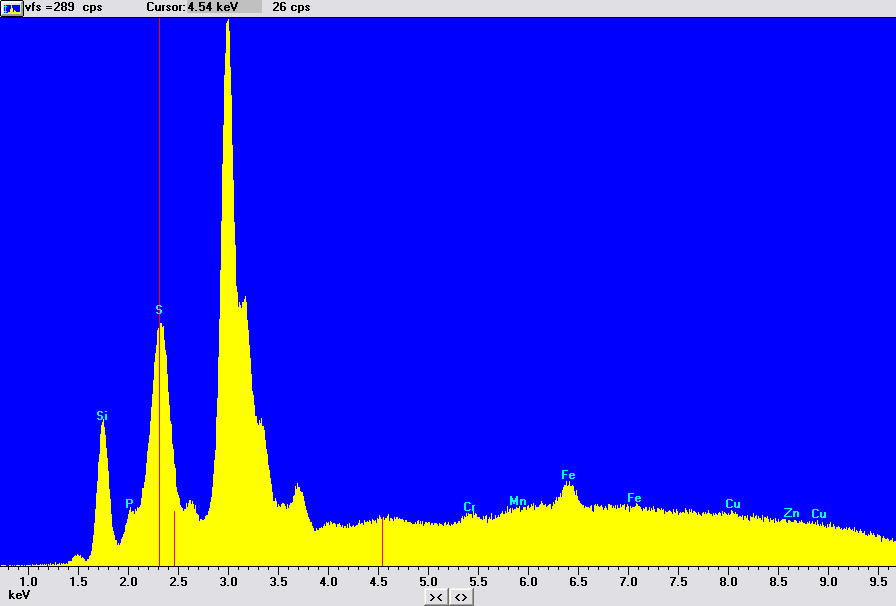

Supplement: Supplemental Information 1 [file peerj-06-5237-s001.zip › RAW/Si X-ray fl.jpg]
